# Standardization of organoid culture for evaluation of melanogenesis induced by UVB, UVA and visible light^☆^^☆☆^

**DOI:** 10.1016/j.abd.2019.06.005

**Published:** 2019-12-06

**Authors:** Thainá Oliveira Felicio Olivatti, Giovana Piteri Alcantara, Ana Cláudia Cavalcante Espósito Lemos, Márcia Guimarães da Silva, Hélio Amante Miot

**Affiliations:** a Faculdade de Medicina de Botucatu, Universidade Estadual Paulista, Botucatu, SP, Brazil; b Department of Dermatology and Radiotherapy, Faculdade de Medicina de Botucatu, Universidade Estadual Paulista, Botucatu, SP, Brazil; c Graduate Program in Pathology, Department of Dermatology and Radiotherapy, Faculdade de Medicina de Botucatu, Universidade Estadual Paulista, Botucatu, SP, Brazil; d Department of Pathology, Faculdade de Medicina de Botucatu, Universidade Estadual Paulista, Botucatu, SP, Brazil

**Keywords:** Melanosis, Organoids, Photobiology

## Abstract

**Background:**

Organoid cultures are primary cultures that maintain architectural characteristics and the relationships between cells, as well as the extracellular matrix. They are alternatives for pathophysiological or therapeutic investigation rather than animal and *in vitro* tests.

**Objective:**

Development of a cutaneous organoid culture model, aiming at the study of radiation-induced melanogenesis.

**Method:**

A validation study, which involved biopsies of the skin of the back of the adult ear. One sample was irradiated with different doses of UVB, UVA, or visible light (VL); the other was maintained in the dark for 72 h. The viability of the tissues was evaluated from the morphological and architectural parameters of the histology, and the expression of the glyceraldehyde-3-phosphate dehydrogenase (GAPDH) gene, by real-time polymerase chain reaction (PCR). The radiation-induced melanin pigmentation was standardized according to the doses of each radiation and evaluated by digital image analysis (Fontana-Masson).

**Results:**

The primary skin culture was standardized at room temperature using DMEM medium. The doses of UVB, UVA, and VL (blue light) that induced differential melanogenesis were: 166 mJ/cm^2^, 1.524 J/cm^2^, and 40 J/cm^2^. The expression of the GAPHD constitutional gene did not differ between the sample of skin processed immediately after tissue collection and the sample cultured for 72 h in the standardized protocol.

**Study limitations:**

This was a preliminary study that evaluated only the viability and integrity of the melanogenic system, and the effect of the radiation alone.

**Conclusions:**

The standardized model maintained viable melanocytic function for 72 h at room temperature, allowing the investigation of melanogenesis induced by different forms of radiation.

## Introduction

The physiopathological investigation and therapeutic responses of certain dermatoses, *in anima nobile*, may be limited by elements related to treatment toxicity, the need for different comparative groups, adherence aspects in therapeutic trials, and the uncertainty regarding the biological response of an intervention (*e.g.*, carcinogenesis).

Animal experimentation increases the range of research possibilities at lower cost, with greater accessibility to subjects and greater exposure control and environmental influence.^1^ However, the physiological responses of animals may not be similar to humans (*e.g.*, melanogenesis), which may lead to misleading conclusions.^2^ In addition, a number of skin diseases lack animal models (*e.g*., melasma), leading to the need for other investigative processes.

Computational simulations of biological phenomena are an option to experiment with living beings, and provide very credible results when simulating interactions between variables widely known from an experimental point of view. Unfortunately, the variables that interfere with cutaneous processes (*e.g.*, melanogenesis) are still not fully elucidated, nor their interaction with other external elements.^3^

Human cell cultures of primary or commercial strains (*e.g*., HaCaT) are widely used in pathophysiological research or for response to specific stimuli. Despite their homology with human tissues, cell line cultures do not replicate the interaction between different cell types in the skin (*e.g*., the epidermo-melanic unit).^4^

Commercial systems with multiple cell cultures (*e.g*., 3D skin: keratinocytes, melanocytes, and fibroblasts) have been propagated as a solution to perceive the interaction between skin cells and are more likely to be related to the tissue structure of the host.^5^, ^6^, ^7^ However, the strains are usually from different donors, with replication patterns somewhat different from what is found in the tissue; also, they do not present endothelium, dendritic cells, or Merkel cells, which, although they are not very numerous in the tissue, may produce some relevant interactions depending on the studied phenomenon. In addition, to date, 3D commercial cultures and commercial skin cell co-cultures do not yet characterize certain diseases.^7^, ^8^, ^9^ Organoid cultures are primary tissue cultures that maintain the architectural characteristics and the main relationships between different cell types, in addition to the extracellular matrix.^10^, ^11^, ^12^ They have the additional advantage that they can be sampled from healthy skin and skin with disease from the same host, favoring the *ex vivo* comparison between the sites.^13^, ^14^ The skin is a relatively viable tissue for organoid culture, with reports of functional maintenance for more than seven days at room temperature.^12^, ^15^, ^16^, ^17^, ^18^, ^19^ Melanogenesis is a complex process, which depends mainly on the action of ultraviolet radiation (UVA and UVB) on the epidermo-melanic unit, with important paracrine regulation, but which also suffers interference from elements of the dermis.^20^, ^21^ Visible light (VL) has been described as a promoter of melanogenesis in high phototypes, and the most biologically active fraction of VL in the skin comprises the blue-violet range (400–500 nm).^22^

This study aimed at the development of an organoid culture model from a skin fragment, in order to study the melanogenesis induced by different forms of radiation.

## Methods

The project was approved by the institution's research ethics committee (No. 2,700,889), and all participants signed the informed consent before inclusion in the study.

A total of ten adult volunteers (>18 years) attended to at the Hospital das Clínicas da Faculdade de Medicina de Botucatu (Brazil), who underwent biopsy of the retroauricular region with a 3-mm punch under local anesthesia (lidocaine 2%), after antisepsis with alcoholic chlorhexidine (2%) and a sterile field. The fragments were sectioned at the level of the deep dermis to avoid large numbers of adipocytes that interfere with complete submergence of the fragment in the culture medium.

The cylindrical fragments were divided longitudinally into two equivalent half-cylinders and stored in the specific DMEM medium (Dulbecco's Modified Eagle Medium – Sigma Inc., United Kingdom),^23^ in a sterile transparent plastic vial, according to the protocol established by Ayres for organoid culture (standardization of viability) at room temperature (20 °C), for posterior dosimetry of the radiation for melanogenesis.^24^ One fragment was irradiated (immediately after collection), and the other, kept in the dark for 72 h.

The fragments were irradiated with increasing doses of UVA, UVB, and VL from artificial sources of UVB (230 μW/cm^2^; source FS72T12/UVB/HO), UVA (1270 μW/cm^2^, source Phillips TL 100W/10R) and LED light (110 mW/cm^2^ in the blue-violet range, source GBRLUX 200 W) standardized at 10 cm. Radiation levels were initiated at 166 mJ/cm^2^, 1.270 J/cm^2^, and 40 J/cm^2^, respectively, and 20% more radiation added to each consecutive sample until differential pigmentation was identified. The absorbance of the walls of the plastic bottles was determined for each form of radiation. A 10 mL volume of the culture medium was used to ensure submergence of the fragment.

Tissue viability was assessed from histological and morphological parameters (H&E), and the quantitative expression of the constitutive GAPDH gene (glyceraldehyde-3-phosphate dehydrogenase) in freshly harvested skins *vs.* those cultured for 72 h was determined by real-time polymerase chain reaction (PCR). The GAPHD gene encodes an elemental protein to perform basic cellular functions (*e.g*., energy metabolism), commonly used as a positive control in real-time PCR reactions, and represents the maintenance of the basal metabolic functions of the cell.

The quantification of the real-time PCR was performed from the amplification of the GAPDH target gene by primer and a validated TaqMan probe. Aliquots of the cDNA (3 μL) extracted from the cutaneous samples were submitted to the PCR reaction using the TaqMan system. Each reaction used 15 μL of the TaqMan Gene Expression Master Mix (Life Technologies – Carlsbad, CA, United States) and 1.0–1.5 μL of the primer set. Amplification was performed on ABI7300 equipment (Applied Biosystems – Carlsbad, CA, United States) using parameters standardized by the manufacturer. The data were processed by the SDS Software System 7300 and the results expressed as threshold cycle (Ct) to determine the expression of the GAPHD cDNA from the number of cycles required to reach the detection limit of the fluorescent signal, which was predefined between 0.2 and 0.3.

The radiation-induced melanin pigmentation was standardized as a function of the doses of each form of radiation as a function of an increase of more than 10% of the pigmentation of the basal layer, evaluated by digital image analysis of the slides stained by Fontana-Masson.^25^ Standardization was completed when the above criteria were met and reproduced.

## Results

The primary skin culture was standardized at room temperature using the DMEM medium. The doses of UVB, UVA, and LV that induced differential melanogenesis were as follows: 166 mJ/cm^2^, 1.524 J/cm^2^, and 40 J/cm^2^, respectively. The absorbance of the plastic bottles was about 50% for all forms of radiation.

**Figure 1:**
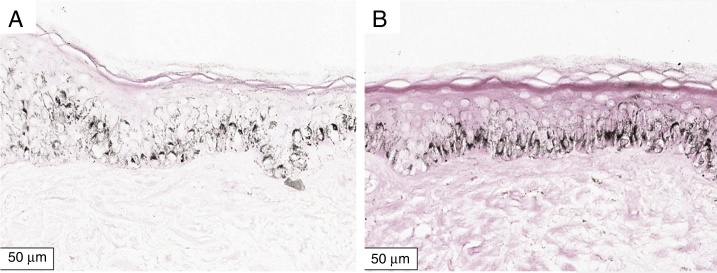
Histological section of organoid culture (72 h) of retroauricular skin (Fontana-Masson, ×400). (A) Culture not irradiated; (B) culture irradiated with UVB, 166 mJ/cm^2^.

**Figure 2:**
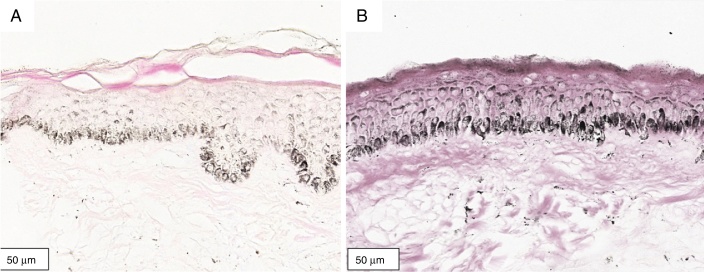
Histological section of organoid culture (72 h) of retroauricular skin (Fontana-Masson, ×400). (A) Culture not irradiated; (B) culture irradiated with UVA, 1.524 J/cm^2^.

**Figure 3:**
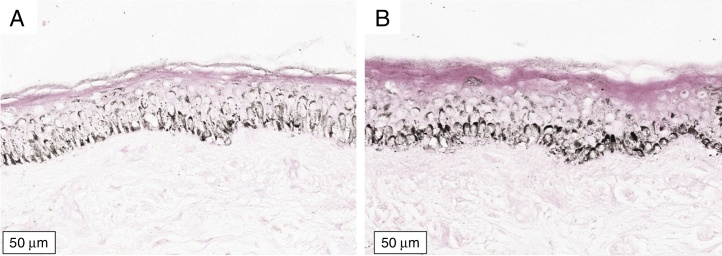
Histological section of organoid culture (72 h) of retroauricular skin (Fontana-Masson, ×400). (A) Culture not irradiated; (B) culture irradiated with visible light, 40 J/cm^2^ (400–500 nm blue-violet).

The melanogenesis of the basal layer could be perceived by Fontana-Masson staining after 72 h of culture (Figure 1, Figure 2, Figure 3).

In the validated protocol, epidermal detachments, pyknosis, and/or necrosis of keratinocytes were not observed. One of the cultures showed contamination after 72 h and was discarded, thus totalling nine patients in the standardization.

**Figure 4:**
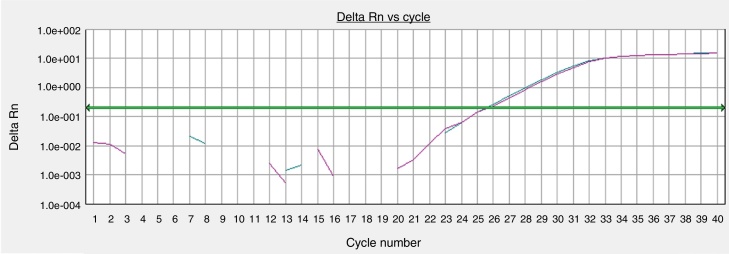
Gene expressions of organoid cultures (72 h) of retroauricular skin. Curves obtained from samples amplified by real-time polymerase chain reaction (PCR) for the endogenous glyceraldehyde-3-phosphate dehydrogenase (GAPDH) gene, whose threshold amplification cycle numbers (Ct) were calculated.

The GAPHD constitutional gene expression was equivalent between the skin sample processed immediately after tissue collection and the sample cultured for 72 h in the standardized protocol (Fig. 4).

## Discussion

The present study validates a reliable low cost alternative to organoid culture of the skin for the study of melanogenesis. The standardized model maintained viable melanocytic function for 72 h at room temperature, allowing the investigation of melanogenesis induced by different forms of radiation. Tissue abundance, easy accessibility for material collection, and architectural and functional maintenance encourage the use of cutaneous organoid cultures in dermatological research.^6^, ^15^, ^18^, ^25^, ^26^

Melanocyte cultures are used for the study of inhibitors of melanogenesis. However, in monocultures, irradiated melanocytes synthesize more pheomelanin than eumelanin, which depends on the interaction with keratinocytes. The investigation of phenomena related to eumelanogenesis is favored, therefore, by organoid models that maintain the influence of different cellular groups.^24^, ^27^

Cutaneous organ cultures using air–liquid media are more commonly used in the study of keratinization, while suspended cultures (as used in this study) allow better perfusion and viability.^12^

Smaller fragments (*e.g.*, 2 mm^2^) tend to have greater viability and architectural preservation due to the more homogeneous imbibition by the culture medium and oxygen distribution to the tissue.^19^, ^26^ In the present study, 3 mm fragments were sectioned in the medium, with adequate preservation of function. One of the fragments that was inadvertently grown without the longitudinal section showed evident nuclear pyknosis in the center of the piece (this case was not included in this study, data not shown).

Modern means of preserving and transporting tissues provide support for the formation of skin banks and also the development of organoid cultures for the standardization of experiments.^28^, ^29^, ^30^, ^31^ The use of bovine serum increases the viability potential of the cultures; however, it compromises the tissue culture as a function of time.^12^ Having the culture at body temperature (37 °C) also increases the viability of the tissue, as well as its metabolic rate, enhancing the exploration of oxidative and mitogenic phenomena such as those involved in carcinogenesis.

Organoid cultures are kept out of interaction with the organism, without thermal, neurological, hormonal, immune, and hypoxic stimuli, which hinder their tissue properties as a function of time.^12^, ^32^ Challenges and stimuli to fragments must be imposed early in order to preserve the credibility of results.

The melanogenesis of the basal layer from the early stimulus was evident in the proposed model. Digital analysis of basal melanogenesis increases the sensitivity of the perception of the phenomenon. Dose–response curves for each type of radiation (alone or in combination) should be explored later, as well as the melanogenic response in melasma.

An additional limitation to the use of whole skin fragments for genomic studies is the heterogeneity of the cells present in the sample, and increased expression of a gene cannot be attributed to a single cell type. For this purpose, primary cell cultures, single-cell, or laser microdissection techniques may be used.^33^

Cutaneous organoid cultures are more available and lower-cost than 3D cultures, presenting greater architectural complexity by contemplating the totality of resident cells (*e.g.*, endothelium, nerves, mast cells) and the extracellular matrix, and they may more adequately represent topographic differences, allowing both genetic and metabolic manipulation, as well as cell isolation; furthermore, they may be derived primarily from dermatosis, with the possibility of comparison with normal skin from the same individual. However, they have less predictable kinetics and cannot be constituted from genetic engineering (*e.g.*, CRISPR, knock-out genes). Both forms of culture have an experimental limitation over time and do not maintain the cutaneous microbiome.^12^ These characteristics should be considered in the choice of experimental models, which should be appropriately standardized and validated for each trial.

The irradiance of sunlight at noon in the interior of Brazil (latitude: 22°53′09″ S; longitude: 48°26′42″ W; altitude: 804 m; 9th of December, 2018) was as follows: 5 mW/cm^2^ (UVB); 1.7 mW/cm^2^ (UVA), and 0.2 W/cm^2^ (blue-violet light). This leads to the hypothesis that less than 15 min of unprotected sun exposure could potentially induce pigmentation of the basal layer, maintaining the limitations of comparison with independent forms of radiation, while the effect of combined radiation or with different photoprotection regimes may present different behaviors.

*In vivo* experimental studies have resulted in effective skin pigmentation from 40 J/cm^2^ in melanodermic individuals (Fitzpatrick IV and V), but not in fair-skinned phototypes. Pigmentation due to UVA1 (340–400 nm) could already be perceived from 5 J/cm^2^ in participants with darker phototypes.^34^

Solar radiation represents a continuum of electromagnetic frequencies that interact with biological tissues and promote diverse stimuli.^35^ For reasons of systematization, the biological effects of radiation are studied from separate wavelengths, but there is some interaction between them.^36^ In the case of cutaneous pigmentation, VL promotes greater intensity and persistence of UVA-induced pigmentation 1.^22^, ^37^

The UVA, UVB, and VL sources of this study promoted independent irradiations, since they did not present significant readings of the other wavelengths (data not shown). This may be relevant because in classical pigmentation studies, such as that by Mahmoud et al., used VL sources that emitted 0.19% UVA and 1.5% infrared; as well as sources of UVA1 that had 0.12% UVA2 and 0.0001% UVB.^34^ The effect of the heterogeneity of the radiation types in the groups makes it difficult to compare them with results from homogeneous sources.

## Conclusion

An organoid culture model was standardized to evaluate the melanogenesis induced by UVB, UVA, and VL.

## Financial support

Fundo de Apoio a Dermatologia de São Paulo – FUNADERSP.

## Authors’ contribution

Thainá Oliveira Felicio Olivatti: Approval of the final version of the manuscript; collection, analysis, and interpretation of data; intellectual participation in the propaedeutic and/or therapeutic conduct of the studied cases.

Giovana Piteri Alcantara: Approval of the final version of the manuscript; conception and planning of the study; collection, analysis, and interpretation of data; participation in the design of the study; critical review of the literature.

Ana Cláudia Cavalcante Espósito Lemos: Approval of the final version of the manuscript; conception and planning of the study; composition of the manuscript; participation in the design of the study; intellectual participation in the propaedeutic and/or therapeutic conduct of the studied cases; critical review of the literature; critical review of the manuscript.

Márcia Guimarães da Silva: Composition of the manuscript; collection, analysis, and interpretation of data; participation in the design of the study.

Hélio Amante Miot: Approval of the final version of the manuscript; conception and planning of the study; composition of the manuscript; collection, analysis, and interpretation of data; participation in the design of the study; intellectual participation in the propaedeutic and/or therapeutic conduct of the studied cases; critical review of the literature; critical review of the manuscript.

## Conflicts of interest

None declared.
